# Supplementary material for: Tuning the Sensitivity and Dynamic Range of Optical Oxygen Sensing Films by Blending Various Polymer Matrices
Source: Biosensors (Basel). 2021 Dec 22;12(1):5. doi: 10.3390/bios12010005 (PMC8773664; doi:10.3390/bios12010005)
Supplement: Supplementary file 1 [file biosensors-12-00005-s001.zip › biosensors-1498920-supplementary.pdf]

biosensors

Supplementary materials

Tuning the Sensitivity and Dynamic Range of Optical Oxygen Sensing Films by Blending Various Polymer Matrices

Kaiheng Zhang [1,2], Siyuan Lu [1,2,*], Zhe Qu [2] and Xue Feng [3]

1 Part Rolling Key Laboratory of Zhejiang Province,School of Mechanical Engineering and Mechanics, Ningbo University, Ningbo 315211, China; geerheng086@gmail.com
2 Institute of Flexible Electronics Technology of THU, Jiaxing 314000, China; quzhe_2013@foxmail.com
3 Department of Engineering Mechanics, Tsinghua University, Beijing 100084, China
* Correspondence: lusiyuan@nbu.edu.cn (S.L.); fengxue@tsinghua.edu.cn (X.F.)

1. Figure S1

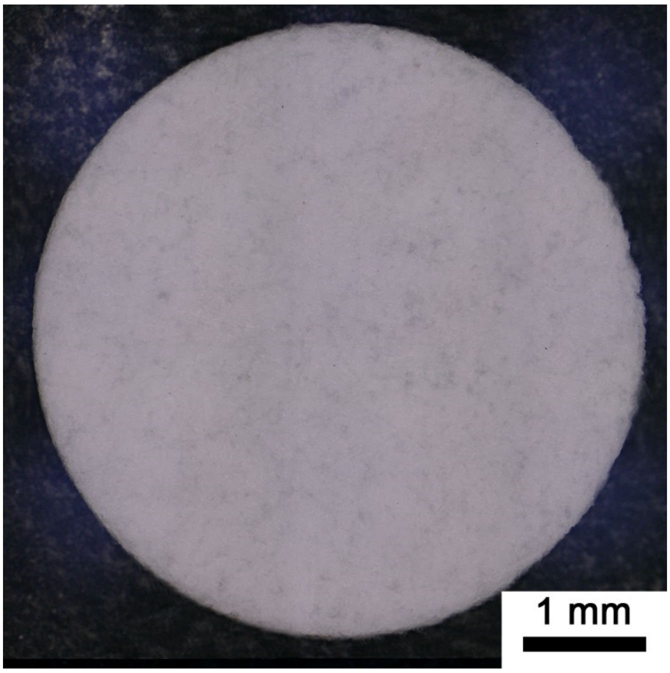

Figure S1. Optical image of microporous filter membrane.

2. Table S1

Table S1. Fitting parameters of hybrid quenching model for experimental sensing films at different temperatures.

Sample \ T (° C)		10	20	30	40	50
SF-EC	A	9.05×10^{-5}	5.18×10^{-4}	2.01×10^{-3}	3.98×10^{-3}	1.15×10^{-2}
	B	0.234	0.283	0.308	0.342	0.185
	C	1.32	1.33	1.48	1.56	2.67
SF-E3P1	A	-1.71×10^{-5}	2.39×10^{-5}	5.38×10^{-5}	9.99×10^{-5}	1.78×10^{-4}
	B	0.0299	0.0328	0.0409	0.0505	0.0623
	C	2.69	2.76	2.75	2.71	2.65
SF-E1P1	A	8.22×10^{-6}	1.81×10^{-5}	1.61×10^{-5}	1.48×10^{-5}	4.35×10^{-6}
	B	0.0230	0.0259	0.0318	0.0374	0.0470
	C	1.69	1.79	1.80	1.86	1.92
SF-E1P3	A	-1.16×10^{-6}	8.85×10^{-6}	2.19×10^{-5}	5.14×10^{-5}	8.60×10^{-5}

	B	0.0194	0.0205	0.0235	0.0276	0.0341
	C	1.15	1.17	1.10	1.22	1.29
SF-PMMA	A	-1.31×10^{-5}	3.76×10^{-6}	1.66×10^{-5}	4.10×10^{-5}	7.71×10^{-5}
	B	0.0168	0.0179	0.0200	0.0226	0.0267
	C	1.02	1.01	1.03	1.05	1.07
